# Optimización de un método de cribado rápido de fármacos en sangre mediante la técnica de cromatografía de líquidos acoplada a espectrometría de masas

**DOI:** 10.1515/almed-2023-0115

**Published:** 2023-10-09

**Authors:** Alba M. Rodrigo Valero, Oscar Quintela Jorge, Begoña Bravo Serrano, Sara Ayuso Tejedor

**Affiliations:** Departamento Madrid, Servicio de Química, Instituto Nacional de Toxicología y Ciencias Forenses (INTCF), Las Rozas, Madrid, España

**Keywords:** cribado, eliminación de fosfolípidos, espectrometría de masas, precipitación de proteínas, sangre

## Abstract

**Objetivos:**

La cromatografía líquida acoplada a la espectrometría de masas ha ganado en popularidad en los laboratorios en los últimos años debido a una mayor especificidad de la técnica, la posibilidad de determinar múltiples analitos en una sola inyección de la muestra, la medición de analitos en una variedad de matrices diferentes y a una drástica reducción de las interferencias analíticas en comparación con el inmunoensayo. El tratamiento y preparación de las muestras biológicas es un proceso esencial cuando éstas han de ser analizadas mediante sistemas cromatográficos. Los principales interferentes en el análisis de las muestras de sangre son los fosfolípidos y las proteínas. El objetivo principal de este estudio es mejorar la sistemática analítica toxicológica en el cribado general de fármacos mediante la técnica LC-MS/MS a través de un nuevo método de preparación de muestras en sangre basado en la precipitación de proteínas y eliminación de fosfolípidos.

**Métodos:**

Se ha evaluado el nuevo método de preparación de muestras en sangre basado en la precipitación de proteínas y eliminación de fosfolípidos mediante la tecnología LC-Q-q-LIT.

**Resultados:**

Se ha determinado el límite de detección, el límite de cuantificación y rango de medición para las 56 moléculas seleccionadas y se han comparado los resultados de once casos con las extracciones e instrumentación tradicionales.

**Conclusiones:**

La metodología propuesta de preparación de muestras en sangre y análisis mediante técnicas más sensibles como LC-Q-q-LIT ha resultado comparable a la metodología tradicional en cuanto a resultados y, ofreciendo, además, una reducción de tiempo y coste.

## Introducción

La cromatografía líquida acoplada a la espectrometría de masas ha ganado en popularidad en los laboratorios clínicos en los últimos 10–15 años [1, 2]. El aumento de su utilidad probablemente se deba a su mayor especificidad como técnica analítica, la posibilidad de determinar múltiples analitos en una sola inyección de la muestra, la medición de analitos en una variedad de matrices diferentes y a una drástica reducción de las interferencias analíticas en comparación con el inmunoensayo [3]. Sin embargo, el inmunoensayo todavía se utiliza habitualmente en muchos laboratorios como prueba de cribado de primera línea, si bien cualquier resultado positivo debe ser confirmado por un segundo método basado en la cromatografía acoplada a la espectrometría de masas [4].

En el ámbito de la toxicología clínica y forense, los compuestos a determinar no se conocen *a priori*. Los inmunoensayos son insuficientes para el estudio de estos casos, ya que están diseñados para el análisis de un número limitado de las sustancias que pueden estar implicadas (o de familias de sustancias) y carecen de la sensibilidad y selectividad necesarias. Por tanto, es necesario aplicar estrategias analíticas de mayor eficacia (Análisis Toxicológico Sistemático, STA) para hacer posible la identificación de cientos de sustancias tóxicas relevantes en muestras biológicas y asegurar así una correcta interpretación de los resultados [5]. Esta estrategia se estructura en dos etapas. En primer lugar, la realización de un cribado de algunas drogas y fármacos en orina y sangre mediante técnicas de inmunoensayo y, en segundo lugar, la confirmación de los resultados obtenidos en el primer nivel y la realización de un cribado ampliado de sustancias no incluidas en dicho nivel. Las principales técnicas analíticas empleadas son: HS-GC-FID, GC-MS, GC-MS/MS, LC-MS/MS incluyendo esta última la espectrometría de masas de alta resolución (LC-HR-MS).

El tratamiento y la preparación de las muestras biológicas es un proceso esencial cuando éstas han de ser analizadas mediante sistemas cromatográficos. Su importancia radica en la necesidad de concentrar o eliminar los distintos componentes de la muestra que pueden interferir, y obtener así una apropiada separación y eficacia cromatográficas. A este respecto la finalidad de la preparación de las muestras, o etapa preanalítica, será concentrar las sustancias objeto de estudio, a la vez que eliminar los posibles interferentes endógenos propios de la matriz biológica. La presencia de proteínas, lípidos, sales y componentes celulares pueden interferir en la medición de la magnitud en estudio, así como condicionar el buen funcionamiento del sistema cromatográfico. Los principales procedimientos de preparación de la muestra son la filtración, la centrifugación, la dilución, la precipitación de proteínas, la extracción líquido-líquido (LLE) y la extracción en fase sólida (SPE), siendo estos dos últimos los más habituales en toxicología clínica para el análisis de sangre y orina [6]. Pese a las ventajas que supone la aplicación de estos procedimientos, la mayoría de ellos pueden dar lugar a una pérdida parcial del componente y, por otro lado, no todos permiten eliminar totalmente los diferentes interferentes de la muestra. Se conoce que los principales interferentes en el análisis toxicológico de las muestras de sangre son los fosfolípidos y las proteínas, por lo que los tratamientos deberían centrarse en reducir su presencia al máximo posible. Es por ello por lo que proponemos realizar durante la etapa preanalítica un procedimiento especial de precipitación de proteínas y eliminación de fosfolípidos como método de preparación de muestras de sangre (suero o plasma), en lugar de mediante las extracciones clásicas SPE y LLE [7]. Dicho método presenta varias ventajas. En primer lugar, disminuiría el tiempo de respuesta, ya que es una técnica de duración más corta en el tiempo. En segundo lugar, también tiene la ventaja de favorecer la detección de más componentes que con las técnicas clásicas, ya que es una técnica menos selectiva. En este caso la menor selectividad radica en que analitos de muy diferente naturaleza química y/o polaridad no se eliminan, como sí podría ocurrir en las etapas de SPE y LLE.

El objetivo principal de este estudio es mejorar la sistemática analítica toxicológica en el cribado general de fármacos mediante la técnica LC-MS/MS a través de un nuevo método de preparación de muestras en sangre basado en la precipitación de proteínas y eliminación de fosfolípidos para el cribado de fármacos. Para su análisis se ha evaluado la capacidad de análisis cualitativo, estudiando el límite de detección, la capacidad de análisis cuantitativo, mediante el análisis de los parámetros habituales de caracterización de linealidad, precisión y exactitud para las moléculas seleccionadas. Asimismo, se ha evaluado su aplicabilidad mediante el análisis de once casos de muestras reales recibidas en el INTCF de Madrid. Los resultados han sido comparados con aquellos obtenidos tras las extracciones e instrumentación tradicionales.

## Materiales y métodos

### Reactivos, soluciones madre y de trabajo

Los patrones internos deuterados obtenidos por Cerilliant^®^ (Round Rock, TX, EE.UU.) fueron los siguientes: clomipramina-d3, ketamina-d4, oxazepam-d5, pseudoefedrina-d3, risperidona-d4, salbutamol-d3, tramadol-d3 y zolpidem-d6. El acetonitrilo LC-MS, el ácido fórmico LC-MS, la solución de ácido fórmico en agua 0,1 % (v/v) y la solución de ácido fórmico en acetonitrilo 0,1 % (v/v) grado LC-MS fueron obtenidas por Fisher Scientific^®^ (Waltham, Massachusetts, EE.UU.). El formiato amónico 1M se obtuvo de Honeywell^®^ (Charlotte, NC, EE.UU.) y el ZnSO_4_ 0,1M de Panreac Química S.L.U (Castellar del Vallès, Barcelona, España). El agua desionizada se obtuvo a través de un filtro Millipak^®^ de Merck-Millipore^®^ (Burlington, MA, EE.UU.) formado por una membrana de filtración de 0,22 µm.

Respecto a las soluciones de trabajo, se preparó la solución de ZnSO_4_ 0,1 M empleada para la lisis celular y la solución de acetonitrilo (ACN) de grado LC-MS acidificada con ácido fórmico (AF) 1 % (v/v) para la precipitación de proteínas. Además, se prepararon la solución de patrones internos deuterados a la concentración conocida de 0,1 mg/L para la cuantificación de los analitos. Por último, también se preparó la mezcla para la reconstitución de la muestras con un 90 % agua y un 10 % ACN, ambas dos acidificadas con un 0,1 % (v/v) de AF.

Se agruparon los 56 fármacos a estudio en función de su grupo terapéutico de la manera que sigue para facilitar el trabajo experimental; grupo 1: clobazam, clotiazepam, flunitrazepam, flurazepam, nitrazepam, prazepam, grupo 2: midazolam, oxazepam, temazepam, tetrazepam, triazolam, zolpidem, grupo 3: bentazepam, clordiazepóxido, diazepam, lorazepam, lormetazepam, nordiazepam, grupo 4: amitriptilina, citalopram, clomipramina, imipramina, paroxetina, sertralina, grupo 5: nefazodona, nortriptilina, reboxetina, trimipramina, venlafaxina, grupo 6: maprotilina, mianserina, trazodona, grupo 7: clonazepam, fenitoína, grupo 8: amoxapina, buflomedilo, clotiapina, codeína, dobutamina, grupo 9: buspirona, metoclopramida, metronidazol, ofloxacina, quetiapina, telmisartán, grupo 10: clozapina, levopromazina, loxapina, risperidona, tioproperazina, tioridazina y grupo 11: 7-aminoclonazepam, N-desalquilflurazepam, zuclopentixol, clorprotixeno y haloperidol. Cada una de las 11 soluciones descritas se prepararon a las concentraciones de 2; 0,2; 0,04 y 0,004 μg/mL. Dichas sustancias se seleccionaron en función de la prevalencia en las intoxicaciones recibidas y de la disponibilidad de patrones de referencia en el laboratorio de Química del INTCF-Madrid.

### Preparación de muestras de sangre

La sangre blanco empleada para la preparación de las muestras es adquirida de forma rutinaria por el INTCF-Madrid del Centro de Transfusión de Sangre de material que por alguna característica no puede ser destinado al uso médico. Se procesa un muestra de sangre blanco junto con el resto de las muestras para verificar la ausencia de fármacos en ella.

La preparación de las muestras de sangre se ha llevado a cabo mediante la siembra en muestras de sangre humana de los analitos seleccionados a las concentraciones de 1,2,5,10,25,50,100,250,350,500,750 y 1,000 ng/mL para los 56 fármacos a estudio a partir de las soluciones anteriormente preparadas.

### Precipitación de proteínas y eliminación de fosfolípidos

La preparación de muestras por precipitación de proteínas y eliminación de fosfolípidos comprende los siguientes pasos, en primer lugar, se lleva a cabo una lisis celular de 100 µL de sangre añadida con 50 µL de la solución de ZnSO_4_ 0,1M. Tras 5 minutos de reposo, se añaden 50 µL de patrón interno y se lleva a cabo la precipitación de proteínas mediante la adición y posterior centrifugado (5 min 14,000 rpm) de 650 µL de la solución 1 % (v/v) AF en ACN. El sobrenadante se carga en la placa Phree Phospholipid de Phenomenex^®^ (Torrance, CA, EE.UU.) y se aplica una presión positiva de 10 psi durante 5 minutos aproximadamente. Seguidamente se evapora a sequedad y se reconstituye con 200 µL de FM. Finalmente se filtra mediante centrifugación (5 min 14,000 rpm) en tubo Eppendorf provisto de un filtro de 0,22 µm de tamaño de membrana de poro.

### Instrumento

El método de LC-MS/MS que se ha empleado consiste en un UHPLC ExionLC acoplado a un Triple Quad 6500+ (QTRAP 6500+) de AB Sciex^®^ (Framingham, MA, EE.UU.) constituido por un triple cuadrupolo híbrido de manera que el tercer cuadrupolo puede actuar como trampa lineal de iones (LC-Q-q-LIT). La función LIT proporciona varios modos de funcionamiento mejorados. Así, los espectros se recogen rápidamente en un periodo de tiempo corto y son significativamente más intensos que los espectros recogidos en un modo de funcionamiento de cuadrupolo estándar comparable, por lo que se aumenta la sensibilidad y se mejora la calidad espectral.

Las muestras se han analizado mediante un nuevo método de escaneo MRM-IDA-EPI (*multiple reaction m* *onitoring-information* *data a* *dquisition-enhanced* *product ion*) que incluye las 56 sustancias a estudio. Para ello, se ha utilizado el software Analyst^®^ y MultiQuant^®^ de Sciex.

#### Parámetros de la cromatografía de líquidos de alta resolución

El cromatógrafo de líquidos incluye un desgasificador, automuestreador, un horno para la columna y dos bombas binarias.

Las FM empleadas son una mezcla de formiato amónico 2 mM en agua con 0,2 % (v/v) ácido fórmico para la FM A y formiato amónico 2 mM en ACN con 0,2 % (v/v) ácido fórmico para la FM B. El gradiente de concentración comienza con un 10 % (v/v) de FM B durante los primeros 0,5 min que aumenta linealmente hasta un 50 % de FM B a los 12 min, un 90 % de FM B a los 14 min hasta un 98 % a los 15,5 min para después descender a las condiciones iniciales a los 17,5 min y mantenerse 2,5 minutos más. La duración total del experimento es de 19,8 minutos y la velocidad de flujo, que se mantiene constante, de 0,3 mL/min.

El volumen de inyección de la muestra es de 2 μL y la temperatura a la que se encuentra el muestreador automático es de 12 °C.

La columna empleada para la separación de los compuestos es la Kinetex 2,6 µm biphenyl 100 Å (100 × 2,1 mm) de Phenomenex^®^ (Torrance, CA, EE.UU.). La temperatura del horno de la columna es de 30 °C.

#### Parámetros de la espectrometría de masas

El espectrómetro de masas consiste en un triple cuadrupolo híbrido con dos bombas de vacío preliminar y una fuente de aire comprimido y nitrógeno acoplado a una fuente de iones IonDrive Turbo V que usa la sonda TurboIonSpray.

Las condiciones de MS son las siguientes: gas de cortina, 35 psi; gas de colisión, intermedio; voltaje de IS, 5500 V; temperatura de la fuente de iones, 450 °C; gas de ionización 1, 50 psi y gas de ionización 2, 60 psi. Se ha trabajado en modo de ionización positivo para todos los compuestos. La trampa de iones trabaja cuando la intensidad de la señal excede de 6000 cuentas por segundo (cps) para iones con una m/z entre 100 y 1250.

Se han utilizado dos transiciones para el análisis de cada analito y una para los patrones internos. La ventana de detección de MRM es de 60 s y el tiempo de escaneo de 0,4 s. La duración del experimento es de 19,8 min y un tiempo de 0,8 s por ciclo. El tiempo de retención, las transiciones y las características específicas de los 56 compuestos como son el potencial de declústering (DP), energía de colisión (CE) y el potencial de salida de la celda de colisión (CXP), están resumidas en la Tabla 1.

**Tabla 1:** LOD, LLOQ, rango de medición, tiempo de retención y condiciones de MS/MS de los 56 analitos analizados por Sciex QTRAP 6500+.

Analito	LOD, ng/mL	LLOQ, ng/mL	Rango medición	Tiempo retención, min	Q1 mass, Da	Q3 mass, Da	DP, V	CE, V	CXP, V	Patrón interno
7-Aminoclonazepam	2	5	5–750	6,41	286,1	222,22	70	29	10	Oxazepam-d5
						121,11	60	35	10	
Amitriptilina	5	10	10–1,000	12,19	278,12	91,15	60	15	10	Zolpidem-d6
						191,14	60	15	10	
Amoxapina	1	2	2–750	10,34	314,1	271,1	60	20	10	Oxazepam-d5
						193,1	60	20	10	
Bentazepam	1	5	5–1000	9,69	297	269,1	60	35	10	Oxazepam-d5
						166,1	60	35	10	
Buflomedilo	1	1	1–1000	7,84	308,2	237,1	60	50	10	Oxazepam-d5
						195,1	60	50	10	
Buspirona	1	2	2–750	9,18	386,3	122,1	41	25	10	Oxazepam-d5
						265,2	41	25	10	
Citalopram	2	10	10–1000	10,37	324,94	109,1	60	35	10	Oxazepam-d5
						262,1	91	27	10	
Clobazam	1	1	1–1000	12,95	301,07	259,18	76	27	10	Risperidona-d4
						224,2	50	46	3	
Clomipramina	2	10	10–1000	13,09	315,16	86,1	60	50	10	Clomipramina-d3
						242,07	60	50	10	
Clonazepam	2	5	5–1000	11,96	315,9	270	36	33	16	Oxazepam-d5
						214,1	100	51	14	
Clordiazepoxido	1	5	5–1000	8,11	300,16	282,1	60	35	10	Oxazepam-d5
						227,18	60	35	10	
Clorprotixeno	2	10	10–750	12,8	316,1	231,1	60	35	10	Risperidona-d4
						221,1	60	35	10	
Clotiapina	1	1	1–750	11,84	344,1	287	60	35	10	Oxazepam-d5
						255,11	60	35	10	
Clotiazepam	2	5	5–1000	13,25	319,1	291,1	60	50	10	Risperidona-d4
						218,17	60	50	10	
Clozapina	1	10	10–750	8,19	327,1	270,1	60	35	10	Risperidona-d4
						192,16	46	57	8	
Codeina	1	2	2–1000	4,6	300,3	215,2	66	33	12	Oxazepam-d5
						165,09	120	55	8	
Desalquilflurazepam	2	5	5–1000	12,03	288,94	226,15	66	50	12	Oxazepam-d5
						140	60	35	10	
Diazepam	1	5	5–750	13,39	284,99	154	60	35	10	Oxazepam-d5
						193,09	60	38	3	
Dobutamina	1	1	1–750	5,93	302,2	137,1	36	31	4	Oxazepam-d5
						107,08	36	31	4	
Fenitoina	10	10	10–1000	10,6	253,1	104,1	100	30	10	Zolpidem-d6
						182,1	61	23	20	
Flunitrazepam	1	10	10–750	12,91	314	268,1	60	35	10	Clomipramina-d3
						239,14	65	49	3	
Flurazepam	1	10	10–750	10,1	388	315,1	71	25	10	Clomipramina-d3
						287,12	50	40	3	
Haloperidol	1	1	1–1000	11,04	376,15	165,12	111	37	22	Oxazepam-d5
						123,05	61	51	14	
Imipramina	1	10	10–1000	11,79	281,2	86,1	76	25	14	Oxazepam-d5
						193,1	76	25	14	
Levomepromazina	1	2	2–1000	12,34	329,2	100,1	60	35	10	Risperidona-d4
						242,15	60	35	10	
Lorazepam	2	5	5–750	11,2	320,95	274,98	60	50	10	Oxazepam-d5
						229,17	50	41	10	
Lormetazepam	1	2	2–750	12,79	334,97	288,97	60	35	10	Oxazepam-d5
						177,19	50	59	8	
Loxapina	1	2	2–500	10,93	328,08	271,07	51	31	16	Zolpidem-d6
						193,19	41	59	10	
Maprotilina	1	10	10–750	11,99	278,2	250,2	60	35	10	Zolpidem-d6
						191,2	100	49	12	
Metoclopramida	1	10	10–750	6,49	300,2	184,1	60	41	12	Oxazepam-d5
						227,2	56	25	10	
Metronidazol	1	2	2–750	3,51	171,81	128,1	60	20	10	Oxazepam-d5
						82,03	60	20	10	
Mianserina	1	2	2–1000	10,48	265,12	208,04	60	35	10	Zolpidem-d6
						193,2	60	35	10	
Midazolam	1	2	2–750	9,87	325,98	291,1	60	35	10	Oxazepam-d5
						249,06	60	35	10	
Nefazodona	1	1	1–750	13,06	470,2	274	60	35	10	Oxazepam-d5
						246,2	60	35	10	
Nitrazepam	2	10	10–1000	11,19	282,1	236,2	60	35	10	Salbutamol-d3
						180,2	126	51	10	
Nordiazepam	1	5	5–750	11,27	270,99	140	60	35	10	Oxazepam-d5
						208,14	60	39	10	
Nortriptilina	1	2	2–750	11,8	264,17	191,16	76	29	14	Oxazepam-d5
						233,12	70	19	14	
Ofloxacina	1	10	10–750	5,5	362,1	261,1	60	35	10	Oxazepam-d5
						318,15	60	35	10	
Oxazepam	5	10	10–750	10,94	287,03	269,2	56	21	32	Oxazepam-d5
						241,11	96	33	14	
Paroxetina	2	25	25–1000	11,37	330,2	192	91	39	16	Zolpidem-d6
						135,07	91	39	16	
Prazepam	1	2	2–750	14,91	325,07	271,14	60	35	10	Risperidona-d4
						165,13	60	35	10	
Quetiapina	1	1	1–750	9,54	384,13	221,14	96	49	12	Oxazepam-d5
						253,06	60	35	10	
Reboxetina	5	10	10–750	10,29	314,17	176,1	126	43	26	Oxazepam-d5
						91,09	40	39	10	
Risperidona	1	5	5–1000	8,49	411	191,3	56	27	12	Risperidona-d4
						163,1	56	27	12	
Sertralina	1	10	10–1000	12,59	306,1	275,1	66	17	14	Zolpidem-d6
						159,02	30	33	10	
Telmisartan	1	5	5–750	12,84	515,2	497,27	100	50	12	Oxazepam-d5
						276,2	60	35	10	
Temazepam	2	10	10–750	12,52	301,2	255,2	101	33	8	Salbutamol-d3
						283,18	76	19	10	
Tetrazepam	5	10	10–750	11,3	288,93	225	60	35	10	Oxazepam-d5
						197,2	106	43	10	
Tioproperazina	2	5	5–1000	11,3	447	141,2	61	55	10	Risperidona-d4
						319,06	61	55	10	
Tioridazina	1	5	5–1000	14,31	371,1	126,2	106	33	10	Clomipramina-d3
						258,11	106	33	10	
Trazodona	1	5	5–750	9,3	372,09	176,13	60	35	10	Zolpidem-d6
						148,04	81	48	10	
Triazolam	1	5	5–750	11,95	343,1	308,2	65	39	3	Oxazepam-d5
						239,2	60	35	10	
Trimipramina	1	2	2–750	12,43	295,2	100,1	60	35	10	Oxazepam-d5
						208,15	60	35	10	
Venlafaxina	1	10	10–750	8,09	278	260,3	60	20	10	Oxazepam-d5
						121,05	60	20	10	
Zolpidem	5	10	10–750	8,5	308,1	235,2	60	20	10	Zolpidem-d6
						263,13	60	20	10	
Zuclopentixol	10	25	25–750	11,8	401,11	356,18	60	20	10	Zolpidem-d6
						271,15	60	20	10	
Clomipramina-d3	–	–	–	13,09	318	89,2	60	35	10	
Ketamina-d4	–	–	–	6,53	241,9	129,1	60	30	10	
Oxazepam-d5	–	–	–	10,94	292,13	246,1	96	21	10	
Pseudoefedrina-d3	–	–	–	3,4	169,1	151	60	20	10	
Risperidona-d4	–	–	–	8,49	415,24	195,14	60	35	10	
Salbutamol-d3	–	–	–	2,54	243,04	151,12	56	27	20	
Tramadol-d3	–	–	–	6,84	268	58	60	35	10	
Zolpidem-d6	–	–	–	8,5	314,32	235,2	61	47	4	

Para la verificación de las sustancias se han evaluado los siguientes parámetros: existencia de pico en el tiempo de retención adecuado, presencia de las dos transiciones MRM por sustancia e identificación del espectro de la sustancia mediante el empleo de una “biblioteca espectral” de compuestos.

### Criterios de aceptación

Para establecer el LOD de cada analito se analizaron los diferentes fármacos a las concentraciones de 1, 2, 5, 10, 25 y 50 ng/mL y se estableció como la menor concentración con una señal: ruido≥3:1. Respecto al LLOQ, se estableció de la misma manera, pero tomando la menor concentración con una señal: ruido≥10:1. Para ello se analizaron por triplicado las muestras.

En cuanto al modelo de calibración, se evaluó la linealidad de todas las sustancias desde 1 ng/mL (CAL 1) a 1000 (CAL 12) ng/mL. Se utilizó el modelo de regresión cuadrática para todas las sustancias excepto para metoclopramida, 7-aminoclonazepam y zuclopentixol, que siguieron un modelo de regresión lineal. Los criterios de aceptación para todas las rectas de calibración que se tomaron, al igual que en estudios similares [8, 9], fueron un coeficiente de determinación (r2) mayor a 0,99, un coeficiente de variación (CV) no mayor a ± 20 % en todos los puntos de la recta y una recta de calibrado formada por al menos siete puntos.

### Análisis de muestras

Para evaluar la aplicación del nuevo método de preparación de muestras se han analizado once casos de muestras de sangre aplicando el método a estudio por el analizador QTRAP 6500+. Dichos casos previamente se habían analizado utilizando los métodos de preparación de muestras tradicionales que consisten como mínimo en una SPE y LLE y por las técnicas de LC-DAD, GC-MS y LC-MS/MS, además del inmunoensayo. Para determinadas sustancias se utilizan también extracciones especiales y/o otras técnicas como GC-MS/MS y LC-HR-MS (Q Exactive Orbitrap). Finalmente se han comparado los resultados de las extracciones y métodos tradicionales con los nuevos propuestos.

## Resultados

Los resultados de los LOD, LLOQ y rango de medición de todas las sustancias analizadas por el QTRAP 6500+ se resumen en la Tabla 1. Todas las sustancias expuestas cumplieron con los criterios de aceptación anteriormente descritos.

Respecto a los once casos seleccionados, diez de ellos eran positivos y uno negativo. Se analizaron las once muestras por el nuevo método, resultando seis positivas y cinco negativas debido a que los fármacos de los cuatro casos positivos no detectados (paracetamol, naproxeno, metamizol y benzoilecgonina) no estaban incluidos en nuestro nuevo método. Respecto a los seis casos positivos, se detectaron las mismas sustancias detectadas por la metodología tradicional a excepción de la sertralina del caso 11 y, en contra posición, se detectó lorazepam en el caso 1 por el nuevo método. En cuanto a su cuantificación, se calculó la concentración mediante la recta de calibrado de cada fármaco y utilizando en cada caso el patrón interno más conveniente, tal y como se detalla en la Tabla 1. Los resultados son comparables a excepción de la quetiapina del caso 1 y el diazepam del caso 11. Los resultados se muestran en la Tabla 2.

**Tabla 2:** Comparación de las sustancias detectadas entre técnicas y métodos.

Núero caso	Metodología tradicional y análisis por inmunoensayo, LC-DAD, GC-MS y LC-MS/MS		Metodología propuesta y analizado por QTRAP 6500+	
1	Lorazepam	ND	Lorazepam	15,4 ng/mL
	Quetiapina	60 ng/mL	Quetiapina	2,6 ng/mL
2	Lorazepam	50 ng/mL	Lorazepam	72,4 ng/mL
5	Codeína	13,3 ng/mL	Codeína	18,1 ng/mL
9	Diazepam	<10 ng/mL	Diazepam	<5 ng/mL
10	Diazepam	40 ng/mL	Diazepam	49,2 ng/mL
11	Metoclopramida	<100 ng/mL	Metoclopramida	40,7 ng/mL
	Haloperidol	8 ng/mL	Haloperidol	1 ng/mL
	Trazodona	300 ng/mL	Trazodona	187 ng/mL
	Diazepam	200 ng/mL	Diazepam	31,9 ng/mL
	Sertralina	100 ng/mL	Sertralina	ND

ND, no se detecta.

## Discusión

La metodología propuesta de preparación de muestras mediante precipitación de proteínas y eliminación de fosfolípidos y análisis mediante técnicas más sensibles como LC-Q-q-LIT para el cribado de 56 sustancias ha resultado comparable a la metodología tradicional ya que se detectan prácticamente las mismas sustancias por ambas dos. Sin embargo, lo consideramos un estudio preliminar ya que se requiere de un proceso de validación más exhaustivo que incluya la evaluación del rendimiento de extracción, efecto arrastre y efecto matriz para todos los fármacos y patrones internos estudiados y de la realización de más estudios para evaluar la cuantificación de sustancias mediante la metodología propuesta. La principal ventaja que ofrece dicha metodología es la reducción de tiempo y coste ya que con sólo una extracción hemos conseguido detectar los compuestos independientemente de su naturaleza con una elevada sensibilidad y selectividad y en un menor tiempo lo que le confiere una gran ventaja en muestras clínicas, sobre todo.

Los factores limitantes de nuestro estudio han sido la no detección de sertralina y el número de sustancias analizadas, pero lo consideramos un buen punto de partida para su futura implementación sobre todo en muestras procedentes de hospitales como son los casos de sumisión química que requieren de rapidez a la vez que de sensibilidad y especificidad.
